# Assessment of *ERBB2* and *TOP2α* gene status and expression profile in feline mammary tumors: findings and guidelines

**DOI:** 10.18632/aging.102079

**Published:** 2019-07-12

**Authors:** Daniela Ferreira, Maria Soares, Jorge Correia, Filomena Adega, Fernando Ferreira, Raquel Chaves

**Affiliations:** 1 CAG - Laboratory of Cytogenomics and Animal Genomics, Department of Genetics and Biotechnology, University of Trás-os-Montes e Alto Douro, 5000-801 Vila Real, Portugal; 2 BioISI - Biosystems and Integrative Sciences Institute, Faculty of Sciences, University of Lisbon, Lisbon 1749-016, Portugal; 3 Research Center for Biosciences and Health Technologies (CBiOS), Faculdade de Medicina Veterinária, Universidade Lusófona de Humanidades e Tecnologias (ULHT), Lisbon 1749-024, Portugal; 4 CIISA - Centro de Investigação Interdisciplinar em Sanidade Animal, Faculdade de Medicina Veterinária, Universidade de Lisboa, Avenida da Universidade Técnica, Lisbon 1300-477, Portugal

**Keywords:** *ERBB2*, *TOP2α*, feline mammary tumors, DNA, RNA, clinicopathological features

## Abstract

In humans, the *ERBB2* gene amplification and overexpression are biomarkers for invasive breast cancer and a therapeutic target. Also, *TOP2α* gene aberrations predict the response to anthracycline-based adjuvant chemotherapy. Although feline mammary tumors (FMTs) are good models in comparative oncology, scarce data is available regarding the *ERBB2* and *TOP2α* status. In this study, and for the first time, the *ERBB2* DNA status and RNA levels of intracellular (ICD) and extracellular (ECD) coding regions were compared with *TOP2α* gene status and expression profile, in samples of FMTs and disease-free tissues from the same animal. Results showed that *ERBB2* and *TOP2α* gene status are highly correlated (r=0.87, p<0.0001, n=25), with few tumor samples presenting amplification. Also, the majority of the FMTs showed *ERBB2* overexpression coupled with *TOP2α* overexpression (r=0.87, p<0.0001, n=27), being the *ERBB2*-ICD and ECD transcripts highly correlated (r=0.97, p<0.0001, n=27). Significant associations were found between *TOP2α* gene status or *ERBB2* and *TOP2α* RNA levels with several clinicopathological parameters. This work highlights the need of experimental designs for a precise evaluation of *ERBB2* and *TOP2α* gene status and its expression in FMTs, to improve their clinical management and to further validate them as a suitable model for comparative oncology studies.

## Introduction

Feline mammary tumors (FMTs) are the third most common cancer in cat, usually highly malignant, infiltrative and metastatic [1, 2], representing a source of aggressive tumor types. FMTs present similar clinicopathological, demographic [3], histopathological [4] and epidemiologic features with human breast carcinomas (HBC) [5], making them excelent models for the study of cancer-related genes [1].

*ERBB2* (Erb-B2 Receptor Tyrosine Kinase 2, also known as HER2) is one of the most studied oncogenes, being considered a breast cancer biomarker (at the gene and protein levels), commonly overexpressed in HBC [6]. ERBB2 is a tyrosine kinase receptor, composed by three different domains: extracellular (ECD), intracellular (ICD) and transmembrane [4, 7, 8]. Studies regarding the intracellular and extracellular domains showed that frequently ERBB2 can be present in truncated forms, being these works highly relevant to predict therapeutic-resistances to ERBB2-targeted drugs (e.g., monoclonal antibodies and small tyrosine kinase inhibitors) [8]. In HBC, the standard method used to evaluate the ERBB2 expression is immunohistochemistry, being the fluorescent *in situ* hybridization used to detect gene amplification [4, 9, 10]. Additionally, the quantification of *ERBB2* RNA levels by real time reverse transcriptase quantitative PCR (RT-qPCR) was also proposed as a potential additional molecular test for the routine diagnosis in HBC and FMT [8, 11, 12]. In cat, ERBB2 is overexpressed in about 30-60% of the FMT [4, 8, 13, 14], but contradictory results have been published. While, De Maria and colleagues [15] reported that ERBB2 is overexpressed in mostly feline mammary lesions, suggesting that FMT is a good model for ERBB2 overexpressing breast tumors with poor prognosis, Soares and co-authors [14] showed that ERBB2 is overexpressed in about 33% of FMTs also indicating that is a suitable model to study ERBB2 positive breast cancers without gene amplification. Santos et al [8] analyzed the ERBB2 protein (both, ICD and ECD) and the RNA levels of the ICD coding region of *ERBB2.* In this work it was reported that *ERBB2* is frequently downregulated in FMT, proposing it as a valuable model for ERBB2 negative breast tumors. Since the above-mentioned studies used different technical approaches and evaluated different tumor samples, further research is needed to clarify the role of the ERBB2 status in the oncogenesis of FMTs towards the validation of new molecular assays and ERBB2-targeted therapies in cat.

Topoisomerase II alfa (TOP2α) is a nuclear enzyme involved in processes such as DNA replication and transcription and chromosome formation, enrichment, and segregation [16], playing a critical role in chromosome instability and tumorigenesis [17]. Also, this protein is suggested as a proliferation marker (as Ki67) due to its overexpression in proliferative cells [18, 19]. TOP2α is increased in around 60% HBCs [20], with triple negative and HER2-positive HBC subtypes presenting higher expression levels of TOP2α than the luminal subtype [21]. Regarding the gene aberrations, in HBC, the amplification of *TOP2α* is correlated with the response to anthracycline chemotherapy and a better outcome of the patient’s survival, independently of its protein expression [22, 23]. TOP2α status has several important implications in breast cancer, however standard tools and cut-off values for estimating TOP2α status have not yet been established [24]. In conclusion, the clinical significance of TOP2α in breast cancer has not yet been clarified [22, 24, 25], being a mandatory research area in this field.

*ERBB2* and *TOP2α* genes are located in the same chromosome in both, cat and human genomes. In HBC, *ERBB2* and *TOP2α* are frequently co-amplified and co-expressed in breast cancer patients [20] and have also been proposed as prognostic biomarkers [26-28]. Furthermore, a positive correlation has been reported between the expression levels of ERBB2 and TOP2α [18, 20], with the TOP2α overexpression being frequently found in ERBB2-positive breast cancer patients[26-28]. Other works, however, suggested that *TOP2α* should be used as an independent breast cancer prognostic and predictive biomarker [22].

In this work, the *ERBB2* and *TOP2α* DNA and RNA status were analyzed and compared between 27 fresh feline mammary tumor (FMTs) samples and disease-free tissues (DFT) collected from the same animals, being these profiles integrated with clinicopathological features. Our results bring new data on the association of *ERBB2* and *TOP2α* DNA and RNA status with oncogenesis and also on *ERBB2* RNA ICD and ECD codifying regions in FMTs.

## RESULTS

### *ERBB2* and *TOP2α* don´t show DNA copy number alterations in FMT

**Figure 1:**
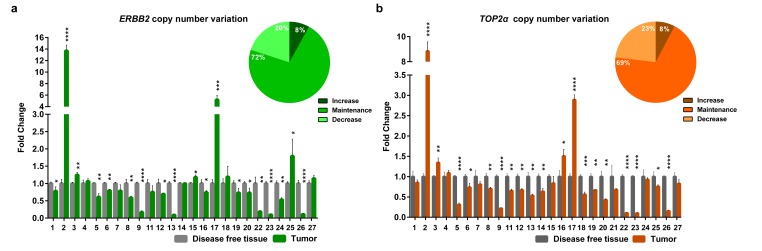
***ERBB2* and *TOP2α* maintain the copy number in feline mammary tumors.** (**a**-**b**) Fold change of *ERBB2* (**a**) and *TOP2α* (**b**) DNA copy number in feline mammary tumors (FMT) analyzed by real-time qPCR and compared with a disease-free tissue (DFT) sample collected from the same animal (control). The percentage of tumors showing an increased, maintained or decreased gene copy number of *ERBB2* (**a**) and *TOP2α* (**b**) is presented in the upper right corner of each graph. Values are mean ± SD of three replicates. *p≤0.05, **p≤0.01, ***p≤0.001, ****p≤0.0001 were determined by Student’s t-test.

**Figure 2:**
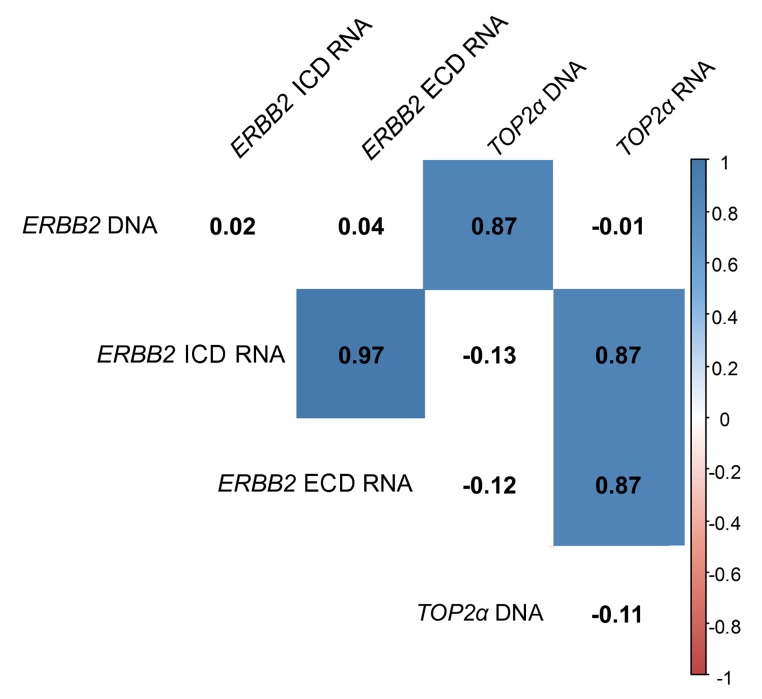
***ERBB2* and *TOP2α* DNA and RNAs correlogram.** Correlation among *ERBB2* and *TOP2α* DNA and RNAs. This correlogram was obtained using the R software. As some analysis presented a different “n”, the data was simultaneously analyzed in GraphPad software and the r-values were corrected by the GraphPad values.

Knowing that *ERBB2* and *TOP2α* genes are located in the same chromosome in human and cat genomes, we analyzed the DNA copy number of *ERBB2* (Figure 1a, Supplementary Table 1) and *TOP2α* genes (Figure 1b, Supplementary Table 2) in a collection of feline mammary tumors (n=27), always in comparison with the disease-free tissue from the same individual. In the majority of the tumors we did not detected *ERBB2* gene amplification (72%) being this result in accordance with previously reported results [8]. It was shown that *ERBB2* gene copy number is increased in 8% (2/25) of the cases and decreased in 20% (5/25) of them (Figure 1a, Supplementary Table 1). The evaluation of the *TOP2α* copy number revealed a similar profile to the observed for *ERBB2*, i.e., only 8% of the tumors presented amplification (2/26 samples) and 23% of the tumors showed a decreased number of copies (6/26, Figure 1b, Supplementary Table 2). Moreover, a strong correlation between the copy number of both genes was found (r= 0.87, p<0.0001, n=25, Figure 2).

### *ERBB2* and *TOP2α* are overexpressed in the majority of the feline mammary tumors

**Figure 3:**
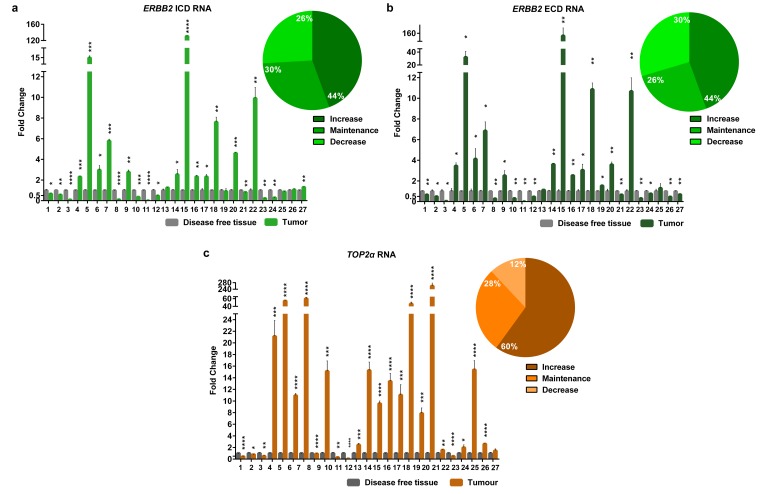
**The RNA levels of the *EBB2* ICD and ECD and *TOP2α* are altered in the majority of the FMTs.** Fold change of *Erbb2* ICD (**a**) and ECD (**b**) RNA regions and *TOP2α* RNA (**c**) quantified by real-time RT-qPCR in FMTs and compared with disease-free tissue collected from the same donor. The percentage of tumors with an increase, maintenance or decrease in the *ERBB2* ICD (**a**), ECD (**b**) and *TOP2α* (**c**) RNA levels is also presented, in the upper right corner of each graph. Values are mean ± SD of three replicates. *p≤0.05, **p≤0.01, ***p≤0.001, ****p≤0.0001 are determined by Student’s t-test.

In this work, the cancer biomarker *ERBB2* and the *TOP2α* gene showed to be correlated at its copy number. So, we decided to analyze both gene expression levels (RNA) in the FMTs collection. Regarding *ERBB2,* the coding regions for both intracellular and extracellular domains (ICD and ECD, respectively) were analyzed, since our group showed that there is a good correlation between the RNA and the protein of the ICD [8]. The results obtained showed that *ERBB2* RNA levels are altered in the majority of FMTs when compared to the disease-free tissue (Figure 3a-b and Supplementary Table 1), with 44% of the tumors showing overexpression of both ICD and ECD transcripts, and different percentages for the downregulation of these two transcripts: 30% for the ECD and 26% for the ICD. Furthermore, the Pearson’s statistical analysis (Figure 2) showed that the expression of both *ERBB2* transcripts (ECD and ICD) is significantly correlated (r=0.97, p<0.0001, n=27). In addition, the quantification of *TOP2α* transcripts revealed its overexpression in 60% of the FMT (Figure 3c, Supplementary Table 2), with *ERBB2* and *TOP2α* expression levels highly correlated (r=0.87, p<0.0001, n=27, Figure 2).

### *ERBB2* and *TOP2α* association with clinicopathological data

Since the animals enrolled in this study were followed up clinically over four years, a statistical analysis was performed on the putative associations between clinicopathological parameters and *ERBB2* and *TOP2α* DNA status and its expression levels (Table 1 and 2). Regarding *ERBB2* (Table 1), a significant association was found between the *ERBB2* RNA levels (both ICD and ECD) and tumor malignancy (p=0.001, n=27) and the higher *ERBB2* expression seems to be related with the lower malignancy grade. In addition, the *ERBB2* RNA levels (both ICD and ECD) were significant associated with the FMT molecular subtypes (p<0.001, n=27), with both luminal A and HER2 subtypes presenting higher *ERBB2* gene expression, and triple negative tumors showing the lowest *ERBB2* RNA levels. Finally, no associations were found between *ERBB2* RNA levels and protein expression, suggesting that a deregulation in transcription and/or translation events of *ERBB2* expression may have occured in these tumors.

**Table 1:** Statistical associations between the *ERBB2* DNA and RNA levels: (ICD and ECD codifying domains) and clinicopathological features, using the one-way ANOVA.

**Clinicopathological features**		***ERBB2* DNA Mean**	***p***	***ERBB2* ICD RNA Mean**	***p***	***ERBB2* ECD RNA Mean**	***p***
*Tumor size*	T1 (< 2 cm)	1.81	0.194 (n=25)	0.71	0.520 (n=27)	0.87	0.470 (n=27)
	T2 (2-3 cm)	0.63		12.73		16.01	
	T3 (> 3 cm)	3.02		2.48		2.67	
*Skin ulceration*	Present	0.49	0.620 (n=25)	7.85	0.976 (n=27)	8.76	0.986 (n=27)
	Absent	1.52		7.29		9.15	
*Sterilized*	Yes	2.00	0.319 (n=24)	11.95	0.333 (n=26)	15.29	0.276 (n=26)
	No	0.83		2.08		2.12	
*Oral contraceptive*	Yes	1.96	0.450 (n=20)	10.32	0.716 (n=21)	12.12	0.839 (n=21)
	No	0.74		5.17		8.70	
*Multiple tumors*	Yes	1.82	0.325 (n=25)	9.85	0.504 (n=27)	11.43	0.608 (n=27)
	No	0.64		3.05		5.20	
*Lymph node with metastasis*	Present	1.68	0.721 (n=24)	3.04	0.468 (n=26)	3.42	0.457 (n=26)
	Absent	1.26		10.47		12.37	
*Tumor stage*	1	2.58	0.608 (n=25)	0.88	0.127 (n=27)	1.07	0.085 (n=27)
	2	0.63		25.46		32.52	
	3	1.53		2.55		2.87	
*Malignancy grade*	1	1.18	0.840 (n=25)	65.82	0.001* (n=27)	77.26	0.001* (n=27)
	2	2.35		1.37		1.40	
	3	1.32		2.83		3.98	
*Necrosis*	Present	1.35	0.766 (n=25)	9.27	0.505 (n=27)	11.52	0.488 (n=27)
	Absent	1.77		1.79		2.27	
*Lymphatic invasion*	Present	3.43	0.067 (n=25)	1.70	0.585 (n=27)	1.64	0.542 (n=27)
	Absent	0.94		8.61		10.82	
*Lymphocytic invasion*	Present	1.40	0.919 (n=25)	3.13	0.222 (n=27)	4.38	0.247 (n=27)
	Absent	1.52		15.74		18.60	
*ki67 index*	High	1.34	0.698 (n=25)	2.75	0.073 (n=27)	3.87	0.085 (n=27)
	Low	1.94		23.37		27.50	
*PR status*	Positive	1.04	0.382 (n=25)	1.98	0.183 (n=27)	2.44	0.161 (n=27)
	Negative	2.04		15.12		18.85	
*ER status*	Positive	0.89	0.586 (n=25)	20.29	0.111 (n=27)	23.84	0.129 (n=27)
	Negative	1.61		2.80		3.97	
*ERBB2 status*	Positive	2.63	0.104 (n=25)	2.79	0.443 (n=27)	4.51	0.513 (n=27)
	Negative	0.77		10.45		12.29	
*Ck5_6 index*	High	1.63	0.682 (n=25)	10.53	0.432 (n=27)	12.38	0.502 (n=27)
	Low	1.15		2.68		4.39	
*Molecular classification*	LB	0.81	0.298 (n=25)	2.21	<0.001* (n=27)	2.74	<0.001* (n=27)
	HER2	5.02		5.18		10.83	
	LBHER2	1.43		1.90		2.14	
	LA	1.18		130.82		153.84	
	TN normal	0.10		0.23		0.32	
	TN basal	0.70		3.95		4.15	

*Indicates *p*≤0.05.

**Table 2:** Statistical analysis between the *TOP2α* gene status and RNA expression with clinicopathological features, using one-way ANOVA test.

**Clinicopathological features**			***TOP2α* DNA Mean**	***p***	***TOP2α* RNA Mean**	***p***
*Tumor size*		T1 (< 2 cm)	1.06	0.159 (n=26)	4.54	0.287 (n=25)
		T2 (2-3 cm)	0.60		38.31	
		T3 (> 3 cm)	2.17		4.87	
*Skin ulceration*		Present	0.46	0.600 (n=26)	59.95	0.473 (n=25)
		Absent	1.12		20.59	
*Sterilized*		Yes	1.39	0.362 (n=25)	13.38	0.480 (n=24)
		No	0.75		29.11	
*Oral contraceptive*		Yes	1.39	0.421 (n=21)	6.76	0.020* (n=19)
		No	0.65		23.40	
*OVH with mastectomy*		Yes	0.74	0.932 (n=12)	35.47	0.588 (n=11)
		No	0.77		0.48	
*Multiple tumors*		Yes	1.27	0.403 (n=26)	26.52	0.621 (n=25)
		No	0.68		15.63	
*Lymph node with metastasis*		Present	1.30	0.587 (n=25)	37.44	0.163 (n=24)
		Absent	0.91		6.79	
*Tumor stage*		1	1.24	0.680 (n=26)	5.68	0.694 (n=25)
		2	0.52		18.09	
		3	1.23		29.02	
*Malignancy grade*		1	0.76	0.895 (n=26)	1.58	0.768 (n=25)
		2	1.46		4.99	
		3	1.04		25.60	
*Necrosis*		Present	1.08	0.976 (n=26)	27.35	0.440 (n=25)
		Absent	1.05		8.85	
*Lymphovascular invasion*		Present	2.56	0.024* (n=26)	56.54	0.102 (n=25)
		Absent	0.72		13.57	
*Lymphocytic invasion*		Present	1.14	0.784 (n=26)	28.89	0.361 (n=25)
		Absent	0.94		7.89	
*ki67 index*		High	1.04	0.871 (n=26)	24.08	0.732 (n=25)
		Low	1.18		14.76	
*PR status*		Positive	0.92	0.597 (n=26)	26.11	0.626 (n=25)
		Negative	1.28		15.16	
*Ck5_6 index*		High	1.22	0.613 (n=26)	28.42	0.512 (n=25)
		Low	0.87		14.21	
*ER status*		Positive	0.77	0.589 (n=26)	12.83	0.627 (n=25)
		Negative	1.18		25.12	
*ERBB2 status*		Positive	1.69	0.139 (n=26)	36.25	0.241 (n=25)
		Negative	0.68		11.10	
*Molecular classification*	LB		0.81	0.282 (n=26)	12.45	0.776 (n=25)
	HER2		3.26		18.33	
	LBHER2		1.01		42.97	
	LA		0.84		0.51	
	TN normal		0.10		8.99	
	TN basal		0.34		22.17	

*Indicates *p*≤0.05

**Figure 4:**
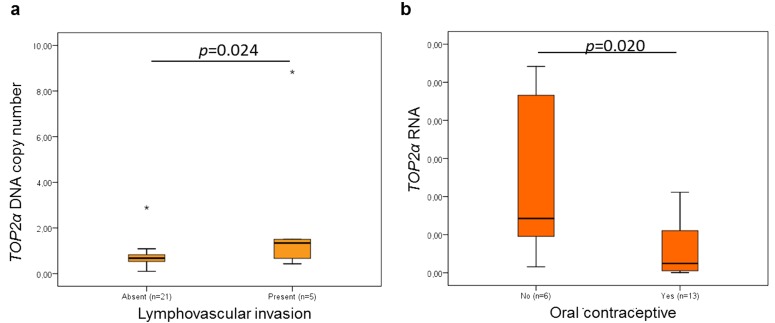
**Statistical associations between *TOP2α* gene status and lymphovascular invasion (a) and between *ERBB2* RNAs levels and oral contraceptive administration (b).** The data are presented as box-plot graphics, showing median, quartiles and extreme values for each category. The *p*-value was obtained by one-way analysis of variance test (ANOVA, Tukey Post Hoc Multiple Comparisons).

Concerning the *TOP2α* DNA and RNA levels and the clinicopathological features (Table 2), a significant correlation between the DNA levels and lymphovascular invasion (Figure 4a, *p*=0.024, n=26) and between the RNA levels and oral contraceptive administration (Figure 4b, *p*=0.020, n=19) was found. The association between the oral contraception and the *TOP2α* RNA levels has not yet been reported, either in humans or in cats, but in the present study, the animals medicated with oral contraceptive showed mammary carcinomas with lower *TOP2α* RNA levels.

## DISCUSSION

The effect of *ERBB2* gene amplification and its concomitant overexpression (in terms of protein levels) on human breast cancer (HBC) patients is well documented. Indeed, *ERBB2* gene amplification [29-31] is considered a biomarker of poor prognosis in HBC, with patients showing a poor disease outcome [29-34], moderately improved by the novel anti-ERBB2 therapies [35]. In parallel, *TOP2α* gene aberrations have been proposed as a biomarker for chromosomal instability [27] and are correlated with the response to anthracycline chemotherapy (or other polychemotherapy regimens) and a better outcome of the patient’s survival, independently of its protein expression [22, 23]. Furthermore, in other works and in HBC patients, the amplification of the *TOP2α* gene has been associated with *ERBB2* gene amplification and related with ERBB2 overexpression [18, 20, 26-28].

Up to now, in HBC, the development of diagnostic tools based on the evaluation of the *ERBB2* RNA levels has been neglected and its usefulness depends on more accurate studies, adequately validated. In this work we evaluated the *ERBB2* and *TOP2α* gene status and RNA levels in a collection of feline mammary tumors using the disease-free tissue sample collected from the same donor as reference and these data were associated with clinicopathological features. It is important to emphasize that the use as a reference sample the disease-free tissue of the same individual, makes our study more robust since each sample is informative about the tumor spontaneously acquired.

It seems that amplification of *ERBB2* gene in ERBB2-positive feline mammary tumors (FMTs) is not frequently found [8, 14]. Additionally, in the FMTs analyzed so far, *TOP2α* gene does not seem to be amplified [14]. A low percentage of tumors presenting co-amplification of *ERBB2* and *TOP2α* genes was found, with the copy number analysis of both genes showing a high correlation, most probably because they are located in the same chromosome [36, 37]. These data is in accordance with other works performed in FMTs and HBCs [14, 18, 20, 38-43].

At this point, it is also important to highlight that the methodologies used to determine the gene status in *ERBB2* is different in HBC and in the FMTs here analyzed. In HBC and following the 2013 ASCO/CAP guidelines (maintained in the 2018 recommendations), the categorization of *ERBB2* is subdivided in three classes by In Situ Hybridization (ISH), being the equivocal cases detected by immunohistochemistry analysis (IHC) resolved by ISH. Thus, in HBC, the ISH test is performed with a dual-probe for *ERBB2* gene and for *CEP17* (a satellite DNA sequence specific for human chromosome 17). In this work, we could not use this method since *CEP17*, a repetitive sequence, is not present in the cat genome. Thus, the use of this probe in FMTs (as reported by [14]) is not conclusive since it cannot exclude cases of polysomy. It is worth mentioning that *ERBB2* gene amplification in HBC is only clinically significant when it is amplified in homogeneous staining regions (HSRs) or as extrachromosomal material [44, 45]. In sum, in the present work (and in our previous one [8]), we are trying to implement a new method for copy number gene determination. However, the data comparison between HBC and FMTs is difficult since the technical principles between both methodologies are different. It will be important, in a near future, to analyze a specific HBC categorized panel following the 2018 ASCO/CAP guidelines to validate the method used in the present work.

Several studies reported the existence of ERBB2 truncated forms, both in cat and in humans, leading in HBC to an ineffective response to anti-ERBB2 therapies (e.g., Herceptin) [8, 46]. These ERBB2 truncated forms are mainly described in HBC patients, with the truncated protein containing only the ICD or ECD or, alternatively, part of both domains [47]. In FMT, a truncated form was described comprising the ICD [4, 8], prompting us to analyzed both RNAs codifying domains of *ERBB2*. With this approach, we were able to evaluate the difference in the transcription of the ECD and ICD, what could be indicative of the presence of a ERBB2 truncated protein. Regarding the results from the quantification of the RNA coding regions for intracellular (ICD) and extracellular (ECD) domains of *ERBB2*, a positive correlation between the expression levels of both RNAs was found (similar quantity of transcripts from both domains), being overexpressed in 44% of the tumors evaluated. Based on our RNA expression results, the following hypothesis are proposed: 1) the FMTs analyzed do not exhibit truncated ERBB2 forms; 2) if there are truncated ERBB2 protein forms, they comprise both domains, even if partially represented; 3) if truncated forms with only one domain are present, they do not result from alternative splicing (in this case there is a post-transcriptional modification, namely, in the translation process, and not in the transcription) [47]. To validate these hypotheses, it would be mandatory to analyze the expression of ERBB2 at protein level, and for both ICD and ECD. However, we were not able to establish a correlation between the levels of the two domains of *ERBB2* transcripts and IHC results (data not shown), in contrast to the results reported by Santos et al. (2013) [8]. Additionally, it was not possible to perform the IHC of the ECD of the ERBB2 protein on these FMTs due to the lack of tumor samples. With these constraints, it is difficult to conclude on the ERBB2 protein *status* in FMT (Table 3). Nevertheless, apart from the fact that the sampling should be larger, some important conclusions can be drawn, since the two *ERBB2* coding domains (ICD and ECD) were quantified, for the first time, by real time RT-qPCR, and a disease-free tissue was used to normalize the data. This last aspect is fundamental, since several works reported that in cat, *ERBB2* gene expression varies in the normal mammary gland, depending on the stage of the estrus cycle, contrasting with the normal human mammary gland [8, 48]. Additionally, the expression of this gene described in normal human tissues disagrees widely between studies, and this is due to its role in normal cells, for instance, the ERBB2 protein is expressed in epithelial cells, particularly those of the secretory epithelia, such as the mammary gland [49]. Thus, we suggest that novel studies should analyze the *ERBB2* expression at RNA and protein levels and, simultaneously, for ICD and ECD coding regions, while the results should be normalize with the values from the disease-free tissue harvested from the same donor, as previous suggested by us [8, 50]. Only with this type of design it will be possible to define the *ERBB2* status (RNA and protein in both domains) in FMTs, contributing to validate the use of cat as a model for ERBB2-positive breast cancer studies.

**Table 3:** *ERBB2* DNA and RNA (ICD and ECD) and *TOP2α* DNA and RNA status: (+ increased, = maintained and – decreased) considering the cut-off 2-fold and ERBB2 protein status following the 2013 ASCO/CAP guidelines (and maintained in the 2018 recommendations) of each of each tumor sample.

	*ERBB2*					*Top2α*	
	DNA	RNA ICD	RNA ECD	CB11/Protein		DNA	RNA
1	=	=	=	1+	negative	=	-
2	+	=	=	2+	equivocal	+	=
3	=	-	-	1+	negative	=	=
4	=	+	+	0	negative	=	+
5	=	+	+	2+	equivocal	-	+
6	=	+	+	1+	negative	=	+
7	=	+	+	2+	equivocal	=	+
8	=	-	-	2+	equivocal	=	=
9	-	+	+	1+	negative	-	+
10		-	-	2+	equivocal		-
11	=	-	-	2+	equivocal	=	-
12	=	-	-	0	negative	=	+
13	-	=	=	1+	negative	=	+
14	=	+	+	1+	negative	=	+
15	=	+	+	1+	negative	=	
16	=	+	+	1+	negative	=	+
17	+	+	+	3+	positive	+	+
18	=	+	+	1+	negative	=	+
19	=	=	=	2+	equivocal	=	+
20	=	+	+	2+	equivocal	-	+
21		=	=	3+	positive	=	=
22	-	+	+	0	negative	-	
23	-	-	-	1+	negative	-	=
24	=	-	=	2+	equivocal	=	=
25	=	=	=	1+	negative	=	+
26	-	=	-	0	negative	-	+
27	=	=	=	0	negative	=	=

In this study, the RNA levels of *TOP2α* were also measured and correlated with the *ERBB2* RNA levels, being overexpressed in 60% of the tumors analyzed, what is in accordance with previous studies [51, 52]. Similarly to the observed for *ERBB2*, no correlation was found between *TOP2α* DNA and RNA levels (results in accordance with [51, 52]). In the future, it will be important to quantify TOP2α protein as there are contradictory published works regarding the clinical significance of the TOP2α expression [22, 24, 25, 51, 52].

From the statistical analysis between the *ERBB2* expression and clinicopathological parameters, we verified that *ERBB2* RNAs levels are negatively correlated with the tumor malignancy grade and, that luminal A and HER2-positive FMT subtypes showed higher *ERBB2* RNAs levels and triple negative FMT subtype the lowest. Regarding the integration of *TOP2α* results with the clinicopathological parameters, a significant association between the *TOP2α* gene status and lymphovascular invasion was found and, between the *TOP2α* RNA levels and oral contraceptive administration. This is the first time that an association between the oral contraception and *TOP2α* RNA levels is reported, but more studies are needed before its use in the veterinary clinical practice.

In summary, and assembling all the data, the following conclusions can be drawn: 1) the co-amplification of *ERBB2* and *TOP2α* genes does not appear to be relevant to their overexpression; 2) other regulatory mechanisms seem to be of major importance in the expression profile of these genes; 3) transcriptional and post-transcriptional mechanisms may be involved in the regulation and expression of these genes in mammary tumors (the co-dysregulation of the aforementioned genes was observed in HBC [20, 39-43], suggesting that FMTs can be used as a cancer model for testing anti-ERBB2 and anti-TOP2α therapies); 4) new experimental designs are needed to define the ERBB2 status towards the validation of FMT as a suitable cancer model and ERBB2 as a valuable biomarker in veterinary medicine. Additionally and in a near future, the accurate evaluation of *ERBB2* expression will have great value improving targeted treatments in cats with ERBB2-positive mammary tumors, especially, with the recent licensing of TKI’s for small animal practice and development of felinized anti-ERBB2 antibodies [53, 54].

## MATERIALS AND METHODS

### Tissue sample collection and characterization

Twenty-seven female cats with spontaneous mammary carcinoma that underwent surgical treatment at the Small Animal Hospital of the Veterinary Medicine Faculty, University of Lisbon, were enrolled in this study. All the owners gave consent for the collection of tumor and disease-free tissue samples, accepting that these might be used for research purposes. In addition, all procedures were carried out in accordance with the EU Directive 2010/63/EU on the protection of animals used for scientific purposes. The tumors were histologically classified according to the World Health Organization (WHO) criteria of dog and cat mammary neoplasms and the malignancy grade was determined using the Elston & Ellis scoring system [55, 56]. The animals’ age ranged from 7 to 17 years, being of different breeds. During the physical evaluation, all the mammary glands and regional lymph nodes were evaluated. When possible, the clinical data and the tumor features were recorded, i.e.: size of the tumor (T1 < 2 cm; T2 > 2 cm and < 3 cm; T3 > 3 cm), animal sterilization, oral contraception, mastectomy accompanied by ovariohysterectomy (OVH), presence of multiple tumors, lymph node with metastasis, necrosis, lymphatic and lymphocytic invasion and skin ulceration. Clinical staging was performed using a TNM system and animals were classified in four stages [57]. All the animals were followed-up after surgery in order to collect data about disease-free survival, overall survival and recurrence type. During the surgical procedure, the excised tumors, normal tissues and were immediately preserved in an RNA stabilization solution (RNA Later Tissue Collection, Ambion) and frozen (at −80°C) to prevent RNA degradation. In addition, a representative area of each mammary carcinoma was formalin-fixed and paraffin embedded for immunohistochemistry (IHC) analysis. ERBB2 (with CB11 antibody for the ICD), Ki-67, PR, ER and CK5/6 immunostaining was performed in accordance to Soares et al. [58] and to the guidelines of the St. Gallen International Expert Consensus panel [59, 60].

### Genomic DNA and RNA isolation

The genomic DNA isolation was performed using 5 mg of each sample (that were cut in small pieces) and the Quick-Gene DNA Tissue Kit S (Fujifilm Life Science) following the manufacturer’s protocol (the tissue lysis step was made by incubation at 70ºC, for 16h). For the RNA extraction were used 60 mg of tissue (that was digested using a cell lysis buffer and a cell disruptor apparatus) and the mirVana™ miRNA Isolation Kit (Ambion, Life Technologies) was performed as described by the manufacturer, being the RNA sample submitted to DNA degradation with the TURBO DNA-free Kit (Ambion, Life Technologies).

### Quantification of *ERBB2* and *Top2α* gene copy number

The *ERBB2* and *TOP2α* gene copy number quantification (primers in Supplementary Table 3) was performed using the standard curve method, as described in Santos et al. [8] and Chaves et al. [61]. The quantification in each DNA sample was obtained by interpolating its CT value against the standard curve. In the PCR reactions were used 10 ng of genomic DNA. The MeltDoctor HRM Master Mix with the SYTO9 dye (Applied Biosystems, Thermo Fisher Scientific) was used in the reactions, following the manufacturer's recommendations. These experiments were performed in a StepOne real-time PCR system (Applied Biosystems, Thermo Fisher Scientific). Briefly, PCR mixtures were exposed to an initial denaturation step at 95°C (10 min), and then to 40 cycles at 95°C for 15 sec followed by 61°C for *ERBB2* or 60ºC for *TOP2α* for 1 min. At the end, a melt curve was performed to evaluate the primers specificity. All reactions were performed in triplicate and negative controls (without DNA) were also included. The StepOne software (version 2.2.2, Applied Biosystems, Thermo Fisher Scientific) was used to create the standard curve (parameters in Supplementary Table 4) and to data analysis. The absolute quantification was transformed in fold-change using the standard curve equation and always in comparison with the respective control sample. A cut-off ≥ 2 times was considered as biologically significant.

### Analysis of RNA expression by real-time RT-qPCR

For *ERBB2* (intracellular and extracellular RNA codifying regions, Supplementary Figure 1) and *TOP2α* RNA quantification (primers in Supplementary Table 3), the standard curve method described by Chaves et al. [61] was used. Standard curve parameters are presented in Supplementary Table 4. For the gene expression quantification, Verso 1-Step RT-qPCR kit, SYBR Green, ROX (Thermo Scientific) was used, following the manufacturer’s recommendations. The absolute quantification of RNA for each sample was obtained by interpolating its CT value against the standard curve. All the PCR reactions were performed in 80 ng of RNA and carried out in a 48-well optical plate (StepOne real-time PCR system, Applied Biosystems, Thermo Fisher Scientific), at 50 °C for 15 min and at 95 °C for 15 min, followed by 40 cycles at 95 °C for 15 sec and at 60 °C for 1 min. Subsequently, a melt curve was performed to evaluate the primer specificity. All reactions were performed in triplicate, and negative controls (without RNA and without Reverse Transcriptase enzyme) were also included in the plate. The data were analyzed using the same parameters and the StepOne software (version 2.2.2, Applied Biosystems, Thermo Fisher Scientific). A cut-off ≥ 2 times was considered as biologically significant.

### Statistical analysis

The statistical software SPSS (Statistical Package for the Social Sciences, version 17.0), the GraphPad Prism 6 (version 6.01) and the R software (The R Foundation for Statistical Computing, 3.3.1 version) were used for the statistical analysis. The Student's t-test (two-tailed) was used for the analysis of the gene copy number variation and transcripts levels between the tumor and the normal samples (real-time qPCR and RT-qPCR results). Statistical associations amongst the clinicopathological parameters were performed using different tests; ANOVA test was performed for analyzing continuous variables with categorical variables and the Pearson’s correlation test to verify the presence of a correlation between continuous variables. When the samples did not present a Gaussian distribution, the values were transformed with the log function in order to normalize the values’ distribution. The correlogram was prepared with GraphPad Prism 6 (version 6.01) and R software (The R Foundation for Statistical Computing, 3.3.1 version). All values are expressed as mean ± SD (standard deviation). The exceptions are the data presented in the box-plot graphics that represents the median, quartiles, and extreme values within a category. In all statistical comparisons, *p* < 0.05 was established as representing significant difference.

### Gene nomenclature

The gene nomenclature used in this work is in accordance with the HGNC-approved official gene symbols [62].

## Supplementary Material

Supplementary File
