# Supplementary material for: Assessment of ERBB2 and TOP2α gene status and expression profile in feline mammary tumors: findings and guidelines
Source: Aging (Albany NY). 2019 Jul 12;11(13):4688–705. doi: 10.18632/aging.102079 (PMC6660035; doi:10.18632/aging.102079)
Supplement: Supplementary File [file aging-11-102079-s001.pdf]

SUPPLEMENTARY MATERIAL

Supplementary Figure

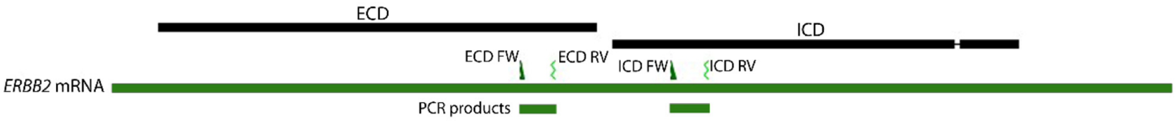

Supplementary Figure 1. Schematic representation of the *ERBB2* ICD and ECD primers location with the correspondence of the proteins domains in the mRNA sequence, resulting from the analysis in Geneious Prime® (2019.1.3).

Supplementary Tables

Supplementary Table 1. ERBB2 DNA and RNA quantification (ICD and ECD transcripts) of each tumor sample using a disease-free tissue collected from de same donor to normalize the data. Values are mean ± SD. A cut-off ≥ 2 times was considered as biologically significant.

	ERBB2 copy number			*ERBB2* ICD RNA			*ERBB2* ECD RNA		
	DFT	FMT	Status	DFT	FMT	Status	DFT	FMT	Status
1	1.00 (±0.04)	0.78 (±0.13)	=	1.00 (±0.10)	0.67 (±3.61x10^{-3})	=	1.00 (±0.02)	0.67 (±0.04)	=
2	1.00 (±0.12)	13.71 (±0.99)	+	1.00 (±1.32x10^{-3})	0.57 (±0.03)	=	1.00 (±0.15)	0.49 (±0.04)	=
3	1.00 (±0.03)	1.25 (±0.08)	=	1.00 (±4.01x10^{-3})	0.09 (±2.12x10^{-3})	-	1.00 (±0.20)	0.04 (±4.58x10^{-4})	-
4	1.00 (±0.10)	1.06 (±0.08)	=	1.00 (±0.01)	2.31 (±0.03)	+	1.00 (±0.27)	3.46 (±0.29)	+
5	1.00 (±0.02)	0.61 (±0.10)	=	1.00 (±0.05)	14.97 (±0.61)	+	1.00 (±0.01)	32.00 (±9.06)	+
6	1.00 (±0.03)	0.80 (±0.03)	=	1.00 (±0.02)	2.98 (±0.42)	+	1.00 (±0.22)	4.13 (±1.00)	+
7	1.00 (±0.0.)	0.79 (±0.17)	=	1.00 (±0.02)	5.79 (±0.17)	+	1.00 (±0.10)	6.85 (±0.86)	+
8	1.00 (±0.12)	0.59 (±0.05)	=	1.00 (±2.27x10^{-3})	0.11 (±4.90x10^{-3})	-	1.00 (±2.54x10^{-4})	0.29 (±0.04)	-
9	1.00 (±0.04)	0.17 (±0.04)	-	1.00 (±0.09)	2.77 (±0.20)	+	1.00 (±3.70x10^{-3})	2.52 (±0.48)	+
10				1.00 (±0.01)	0.36 (±4.41x10^{-4})	-	1.00 (±0.08)	0.32 (±0.03)	-
11	1.00 (±0.12)	0.75 (±0.18)	=	1.00 (±0.01)	0.01 (±2.73x10^{-3})	-	1.00 (±0.05)	0.01 (±7.19x10^{-6})	-
12	1.00 (±0.13)	0.70 (±0.02)	=	1.00 (±0.08)	0.48 (±0.06)	-	1.00 (±0.02)	0.46 (±0.04)	-
13	1.00 (±0.06)	0.09 (±0.02)	-	1.00 (±0.14)	1.26 (±0.04)	=	1.00 (±0.05)	1.13 (±0.01)	=
14	1.00 (±0.03)	1.00 (±0.03)	=	1.00 (±0.09)	2.53 (±0.49)	+	1.00 (±0.10)	3.62 (±0.07)	+
15	1.00 (±0.03)	1.18 (±0.06)	=	1.00 (±0.03)	130.82 (±0.06)	+	1.00 (±0.17)	153.84 (±19.48)	+
16	1.00 (±0.11)	0.75 (±0.05)	=	1.00 (±0.05)	2.33 (±0.12)	+	1.00 (±0.08)	2.54 (±0.03)	+
17	1.00 (±0.11)	5.20 (±0.78)	+	1.00 (±0.19)	2.32 (±0.22)	+	1.00 (±0.18)	3.05 (±0.57)	+
18	1.00 (±0.14)	1.19 (±0.31)	=	1.00 (±2.82x10^{-3})	7.62 (±0.47)	+	1.00 (±0.08)	10.86 (±0.63)	+
19	1.00 (±0.09)	0.73 (±0.13)	=	1.00 (±0.09)	0.90 (±0.28)	=	1.00 (±0.02)	1.55 (±0.08)	=
20	1.00 (±0.06)	0.73 (±0.13)	=	1.00 (±0.06)	4.60 (±0.06)	+	1.00 (±0.21)	3.59 (±0.25)	+
21				1.00 (±0.02)	0.82 (±0.01)	=	1.00 (±0.03)	0.67 (±3.48x10^{-3})	=
22	1.00 (±0.18)	0.19 (±0.02)	-	1.00 (±0.16)	9.92 (±1.05)	+	1.00 (±0.18)	10.67 (±1.31)	+
23	1.00 (±0.07)	0.10 (±2.60x10^{-3})	-	1.00 (±0.04)	0.23 (±8.06x10^{-3})	-	1.00 (±0.07)	0.32 (±0.01)	-
24	1.00 (±0.08)	0.54 (±0.05)	=	1.00 (±0.05)	0.29 (±0.04)	-	1.00 (±0.05)	0.76 (±0.02)	=
25	1.00 (±0.05)	1.79 (±0.49)	=	1.00 (±0.04)	0.87 (±0.05)	=	1.00 (±0.21)	1.30 (±0.43)	=
26	1.00 (±0.09)	0.11 (±0.02)	-	1.00 (±0.05)	1.07 (±0.10)	=	1.00 (±6.48x10^{-4})	0.46 (±0.04)	-
27	1.00(±0.08)	1.14 (±0.09)	=	1.00 (±0.01)	1.31 (±0.02)	=	1.00 (±0.03)	0.69 (±0.01)	=

Supplementary Table 2. *TOP2α* DNA and RNA quantification of each tumor sample using a disease-free tissue collected from de same donor to normalize the data. Values are mean ± SD. A cut-off ≥ 2 times was considered as biologically significant.

	TOP2α DNA			*TOP2α* RNA		
	Disease-free	Tumor	Status	Disease-free	Tumor	Status
1	1.00 (±0.14)	0.85 (±0.07)	=	1.00 (±3.00x10^{-3})	0.44 (±0.04)	-
2	1.00 (±0.08)	8.83 (±0.75)	+	1.00 (±0.09)	0.79 (±0.03)	=
3	1.00 (±0.03)	1.34 (±0.12)	=	1.00 (±0.08)	0.53 (±0.07)	=
4	1.00 (±0.11)	1.09 (±0.06)	=	1.00 (±0.04)	21.14 (±2.72)	+
5	1.00 (±0.03)	0.31 (±0.04)	-	1.00 (±0.05)	54.14 (±1.21)	+
6	1.00 (±0.06)	0.74 (±0.10)	=	1.00 (±0.08)	10.95 (±0.34)	+
7	1.00 (±0.16)	0.81 (±0.07)	=	1.00 (±0.01)	59.95 (±3.45)	+
8	1.00 (±0.08)	0.70 (±0.04)	=	1.00 (±7.50x10^{-5})	0.92 (±0.01)	=
9	1.00 (±0.05)	0.22 (±0.02)	-	1.00 (±0.11)	15.13 (±1.77)	+
10				1.00 (±0.17)	0.31 (±0.01)	-
11	1.00 (±0.07)	0.65 (±0.04)	=	1.00 (±0.05)	0.06 (±3.01x10^{-3})	-
12	1.00 (±0.07)	0.67 (±0.04)	=	1.00 (±0.04)	2.46 (±0.23)	+
13	1.00 (±0.11)	0.53 (±0.03)	=	1.00 (±0.03)	15.28 (±1.42)	+
14	1.00 (±0.07)	0.63 (±0.08)	=	1.00 (±0.08)	9.56 (±0.45)	+
15	1.00 (±0.06)	0.84 (±0.17)	=			
16	1.00 (±0.15)	1.50 (±0.17)	=	1.00 (±0.01)	13.39 (±1.35)	+
17	1.00 (±0.07)	2.89 (±0.13)	+	1.00 (±0.02)	11.05 (±1.77)	+
18	1.00 (±0.06)	0.56 (±0.06)	=	1.00 (±0.01)	46.58 (±4.58)	+
19	1.00 (±0.07)	0.67 (±3.00x10^{-3})	=	1.00 (±0.09)	7.88 (±0.93)	+
20	1.00 (±0.13)	0.43 (±0.03)	-	1.00 (±0.11)	260.11 (±21.79)	+
21	1.00 (±0.28)	0.68 (±0.03)	=	1.00 (±0.13)	1.58 (±0.06)	=
22	1.00 (±0.05)	0.10 (±0.01)	-			
23	1.00 (±0.02)	0.10 (±0.02)	-	1.00 (±0.05)	0.51 (±0.01)	=
24	1.00 (±0.17)	0.93 (±0.05)	=	1.00 (±0.06)	1.98 (±0.47)	=
25	1.00 (±0.12)	0.76 (±0.04)	=	1.00 (±0.03)	15.38 (±1.57)	+
26	1.00 (±0.01)	0.16 (±0.01)	-	1.00 (±0.01)	2.61 (±0.03)	+
27	1.00(±0.10)	0.83 (±0.10)	=	1.00(±0.13)	1.45 (±0.31)	=

Supplementary Table 3. Sequence of the primers used in this work.

	Forward	Reverse
ERBB2 DNA	GAGTGCGGTAAGACAGGGAG	GTCTGCACAAGTCCGAGAT
ERBB2 ICD RNA	GGTGTTCTCGGACATGGTCT	CTCCCAAAGCCAACAAAGAA
ERBB2 ECD RNA	AGGAATGCCGAGTATTGCAG	GGTCCTTGTAGTGGGCACAG
TOP2α	ACAGGTGGTCGAAATGGCTA	ATTCTCTACTGGCTGTTTCC

Supplementary Table 4. Standard curve parameters.

	R^2	Efficiency (%)
ERBB2 DNA	0.996	97.99
TOP2α DNA	0.998	100.72
ERBB2 ICD RNA	0.99	98.11
ERBB2 ECD RNA	0.99	102.17
TOP2α RNA	0.999	96.693
